# Computing and Oral Health: Mobile Solution for Collecting, Data Analysis, Managing and Reproducing Epidemiological Research in Population Groups

**DOI:** 10.3390/ijerph17031076

**Published:** 2020-02-08

**Authors:** Nilton Vale Cavalcante, Ary Henrique Oliveira, Bruno Vinícios Cunha de Sá, Glenda Botelho, Tiago Ricardo Moreira, Glauce Dias da Costa, Rosangela Minardi Mitre Cotta

**Affiliations:** 1 Department of Medicine, Federal University of Tocantins, Palmas, TO 77090-001, Brazil; vale@uft.edu.br; 2 Department of Computer Science, Federal University of Tocantins, Palmas, TO 77090-001, Brazil; bcunha@id.uff.br (B.V.C.d.S.); glendabotelho@uft.edu.br (G.B.); 3 Department of Medicine and Nursing, Federal University of Viçosa, Viçosa, MG 36570-900, Brazil; tiago.ricardo@ufv.br; 4 Department of Nutrition and Health, Federal University of Viçosa, Viçosa, MG 36570-900, Brazil; glauce.costa@ufv.br (G.D.d.C.); rmmitre@ufv.br (R.M.M.C.)

**Keywords:** epidemiological surveys, oral health, computer software, DMF index, computing applied to dentistry

## Abstract

Epidemiological inquiries study and evaluate the health status of the population. For dental caries, the World Health Organization (WHO) recommends the DMFT and DMFS indexes, which represent the sum of the decayed, missing and filled teeth, divided by the population studied. Traditionally these surveys are conducted using cellulose paper sheet. This study describes the development and presents the field performance of NutriOdonto, a software created for an Oral Health Survey carried out in 2018 and 2019 involving 2578 students from the municipal schools of Palmas/TO, located in the Brazilian Amazon region. This is a descriptive, applied research on the development of a software for the collecting, analysis, management and reproducibility of oral health epidemiological research. A software applied to the collecting, analysis and formation of the database was developed through the information obtained from the questionnaires applied to the participants of the study and the completion of the electronic oral examination form. Recent Information and Communication Technologies (ICT) are intelligently configured to create models and mobile applications (Apps) that can be useful to manage health issues, thus broadening the perspective of service provision in this sector. Some of these mobile devices, tablets and smartphones are being developed to generate information, for collection, recording, storage and analysis of oral health epidemiological research data. NutriOdonto contributed to the rapid collection, recording and storage of information, in the construction of the database and its analysis. Replacing paper forms with electronic forms minimized possible typos, reduced the use of cellulose paper and the financial costs, among other things. This software can contribute to decision making by managers and professionals and to improving the planning and implementation of actions in health promotion and oral disease prevention.

## 1. Introduction

In 2010, oral problems affected 3.9 billion people worldwide and tooth decay in permanent teeth was the most predominant condition assessed for the entire 2010 Global Burden of Disease (GBD) study, with a global prevalence of 35.4%. in all ages combined, followed by periodontitis with 10.8% [1]. The World Health Organization (WHO) considers that public health problems associated with oral diseases are a major burden in countries around the globe. Therefore, conducting epidemiological surveys to determine the condition of oral health and the need for intervention in communities, becomes an essential part of the daily lives of health managers and other professionals responsible for oral health programs [2].

In order to know and compare the experience of dental caries in population groups, the WHO recommends the ceo and CPO indexes, respectively for deciduous and permanent dentition. These indexes represent the sum of the number of decayed, missing and filled teeth divided by the number of individuals searched [3,4]. Traditionally, when conducting the ceo and CPO, appropriate and standardized paper records are used to register oral health epidemiological assessments [5] however, in the last decades, digital technologies have been increasingly used in the health sector, collaborating in the diversification and flexibility of the activities produced by students, researchers, professionals and managers of this area [6]. Thus, in an irreversible process, paper records are being replaced by electronic records, enabling numerous advantages provided by this means and providing opportunities for professionals and institutions, according to technological evolution, to a further adoption of these records in the most diverse activities [7]. Health and Technology are recurrent themes both in our daily lives and in the various domains of knowledge in today’s society [8]. The development of new information technology (IT) associated with rising health costs, has led to a new frontier area: the electronic Health (eHealth), defined as the use of information and communication technologies to offer and improve health services [9].

In this sense, the use of mobile technologies to support the achievement of health objectives. has the potential to influence and transform health services provision worldwide, and a powerful combination of factors is promoting this change. This includes rapid advances in mobile technologies and applications, an increase in new opportunities for integrating mobile health into existing healthcare services, and the continued growth in cell phone network coverage [10]. This opens new perspectives for the collection of biological, behavioral and emotional data, including therapeutic interventions. Among the potential applications of this type of technology are: health promotion and community mobilization actions; health education campaigns; epidemiological surveillance and monitoring; development of decision making support systems [9].

Research on the development of mobile applications for use in dentistry and related fields have been increasingly published in national and foreign literature. In a single integrative literature review by Barra [11] on methods for developing mobile applications in health, 28 studies for the identification of such methods were published between 2012 and 2016. In the field of dentistry, new tools also known as Information and Communication Technologies (ICTs) have been contributing to the analysis of the determinants of dental caries, and in the decision making and its operational characteristics [12].

The city of Palmas is less than thirty years old. It was built to be the capital of the State of Tocantins, created by the Brazilian Federal Constitution of 1988 [13]. According to the local Municipal Governance on Oral Health, Palmas has never conducted an epidemiological survey of dental caries [14]. Its epidemiological data are obtained from the National Oral Health Surveys, carried out by the Ministry of Health. The CPO Index of Palmas in 2010 at the age of 12 years old was 2.35 [15].

This way, the objective of this study was to describe the construction process and present the field performance of NutriOdonto, a software created for conducting an Epidemiological Caries Survey also conducted in the city of Palmas/TO, Brazil, involving 9th grade Elementary School students, enrolled in schools of the municipal education system, linked to the School Health Program (PSE).

## 2. Materials and Methods

The activities carried out in this work were approved by the Research Ethics Committee (CEP) of the Federal University of Viçosa (UFV), CAAE number 87863218.7.0000.5153 with opinion number 2.760.864. It is noteworthy that the actions were carried out based on the goals set by the United Nations (UN) Sustainable Development Goals (SDGs), especially those related to SDG3, which includes ensuring a healthy life and promoting well-being for all, at all ages, and the SDG17, which aims to strengthen the means of implementation and revitalization of the global partnership for sustainable development. The project was made possible due to the construction of a partnership between the Municipal Health Secretariat of Palmas-TO, the Municipal Secretariat of Education of Palmas-TO and the Federal University of Tocantins (UFT).

**Figure 1:**
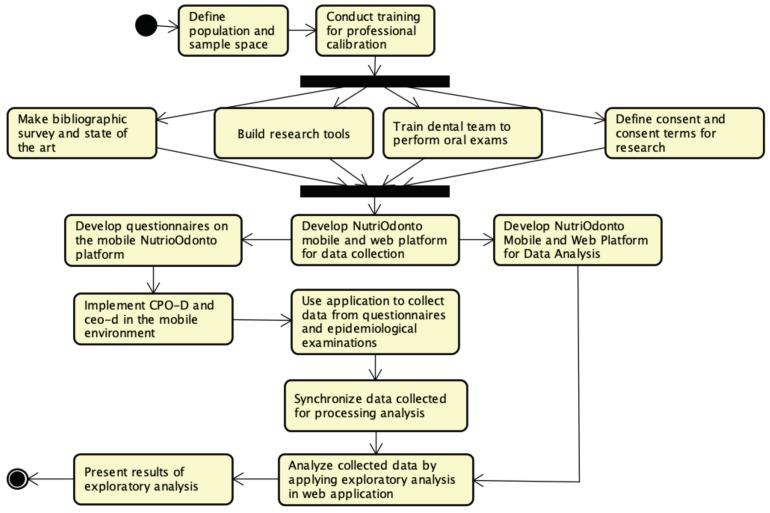
Steps to the development of the NutriOdonto environment. Source: Author’s own creation.

The mechanism for collecting, storing and processing epidemiological analyses is a computational environment composed of two main modules: a mobile application used for collecting and storing digital data in remote locations, and a data synchronization environment and analyses processing at a central server on the web. The computational environment called NutriOdonto manages the information collected in electronic records, based on those proposed by Klein and Palmer in 1937 to obtain the indexes ceo (deciduous dentition) and CPO permanent dentition), still recommended by the World Health Organization to conduct epidemiological surveys on dental caries [4]. Its construction was idealized from a well-elaborated bibliographic survey and the observation of the training and calibration of the participants to perform the research and the oral examinations. Figure 1 presents the steps taken to develop the NutriOdonto environment.

Initially, it was necessary to define the size of the samples treated by the evaluation instrument as well as the analysis plans employed by the application. It was defined that the survey of dental caries would be performed on the public consisting of students of municipal schools from Palmas-TO, including both genders, with ages ranging largely from 12 to 19. In the 2017 school year, the Palmas-TO municipal education network had approximately 16,321 enrolled students, of which 2014 were in the 9th grade of elementary school [16]. This population was invited to participate in the survey, encompassing all students from 29 schools with more than 15 students enrolled in that grade.

In 2017, the researcher and dental surgeons of the Oral Health Teams (ESB) from the Primary Health Care of the municipal health network of Palmas-TO, were trained to carry out the epidemiological survey. In that year, they organized workshops and conversation roundtables that aimed to detail the operationalization of the work stages, clarify each participants attributions and discuss the theoretical and practical aspects of the indexes that were to be used. In this sense, the WHO establishes standards of diagnostic criteria for oral health epidemiological studies and recommends that all examiners involved in these studies should previously participate in a calibration step, a training which implies the repetition of examinations in the same people by the same examiners, or by the same examiner at different times, in order to select the professionals who can reproduce in a statistically reliable manner the criteria adopted in the study in order to reduce the interpretation discrepancies in the diagnoses [4,5].

The following steps were composed of a literature review, a definition of research instruments and team training to perform oral examinations. From this point on, the data collection process was initiated, starting with oral exams, followed by the application of a Self-Applicable Structured Questionnaire for the socioeconomic characterization of the students and the application of the Self-Applicable Structured Questionnaire for the socio-demographic characterization of the 26 schools involved in the study survey, answered by the directors of these institutions. These instruments were adapted to the local reality, based on the same ones used in the National School Health Survey (PeNSE), developed in Brazil in 2015 [17]. The Terms of Free and Informed Consent (TCLE), as well as the Terms of Consent (TA) were also defined.

The NutriOdonto computational environment was developed from the set of information regarding the flow of processes for an oral health epidemiological survey. The development of two modules has been selected: a mobile application for remote data collection and storage and a web application for centralized database management and analyses processing—based on synchronized data from mobile applications. In this context, the traditional paper pulp sheets were replaced by electronic forms and data were collected using smartphones and tablets. With data stored electronically in databases, analyses processing was performed instantly as questionnaires were answered and synchronized between mobile applications and the web module. The following sections will present the characterization of the NutriOdonto environment.

### 2.1. NutriOdonto Environment Architecture

**Figure 2:**
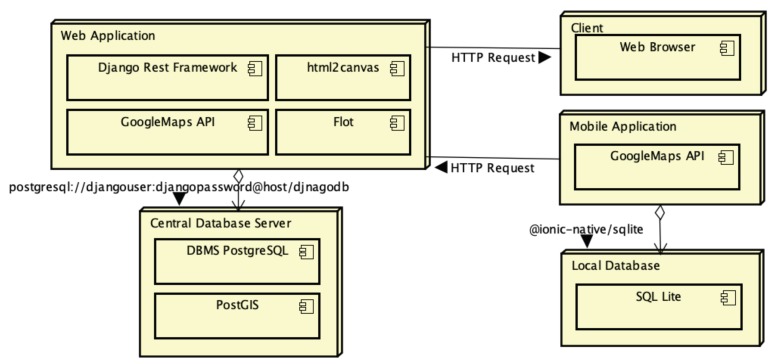
Implementation design of the NutriOdonto environment. Source: Author’s own creation.

NutriOdonto is a web application deployed under an application server that offers a set of features for managing the epidemiological inquiry through a web portal that can be accessed by multiple users via a web browser that receives requests under Hyper Text Transfer Protocol (HTTP) and Hyper Text Transfer Protocol Secure (HTTPS). NutriOdonto has a mobile app designed for data collecting activities in remote locations with no internet access. Therefore, the mobile app stores the data in a local database on the device temporarily, be it a tablet or a smartphone, and when it finds an internet connection, it synchronizes data from the local database with the central database. Figure 2 presents the components that form the NutriOdonto computational architecture. In the context of the web application, NutriOdonto was designed under the three-tier client-server architecture, where users who access the environment via web browser are customers of the services offered by NutriOdonto, which is implemented on the application server. The NutriOdonto service is characterized as a database server client that has been deployed on a separate server to maintain the security of stored data. NutriOdonto has an application in the mobile application version, used to perform epidemiological data collection in the field. The application temporarily stores data collected on the mobile device during field data collection activities and then synchronizes the field data collected, with the central application server maintaining thus the information updated. Nutriodonto has six modules:

- Portal module: containing the functions for recording information about project execution, such as news about the execution of epidemiological survey actions, documents, photos and videos.
- Data collection module: which concentrates the data collection functions on health actions, organized in the form of campaigns, characterization of school units, principals and students, as well as the application of questionnaires and oral examinations.
- Administration module: deals with security mechanisms of the computing environment, such as access control mechanisms and encryption of information stored in the database.
- Integration module: allows data export to external analysis tools in the CSV and XML formats.
- Analysis module: implements the information analysis mechanisms.
- Geographic module: performs data management through a geographic information system.

**Figure 3:**
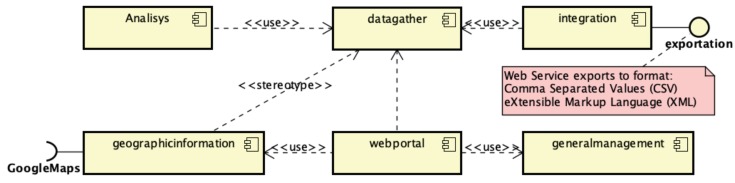
Main modules of the NutriOdonto computing environment. Source: Author’s own creation.

The component diagram in Figure 3 shows the organization of the NutriOdonto modules with the dependencies of component use. The web portal component obtains the data from a big part of the components in order to assemble the site presentation information. The integration component has a communication interface that enables external systems to acquire stored database information through a connection token, preserving the security guidelines of information stored in the system. The datagather component is the module that possesses all data collection mechanisms of the system, so the other modules look for information contained under its management. The geographic information component uses GIS service APIs applications, such as Google Maps to manage geographic data.

### 2.2. NutriOdonto Functional and Non-Functional Requirements

**Figure 4:**
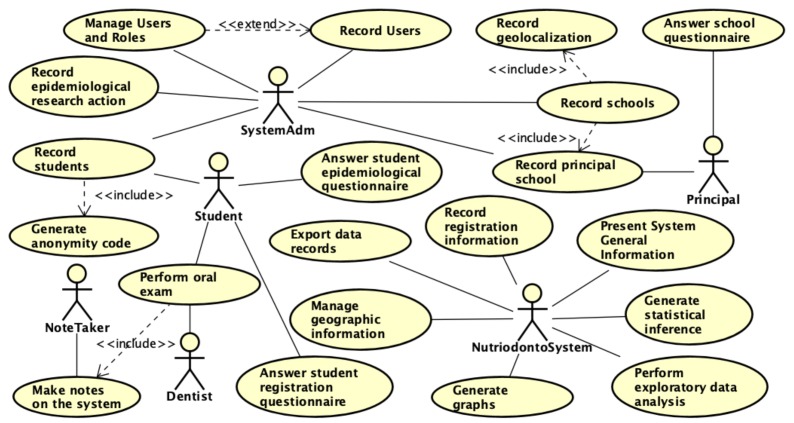
Use case diagram with the functional requirements raised to build the NutriOdonto computing environment. Source: Author’s own creation.

The use case diagram in Figure 4 below, summarizes the key features implemented in the computing environment. These are the essential functions for carrying out the activities of collection, storage and analysis of the NutriOdonto system. The functions were related to the modules presented in Figure 3, in a way that enables the monitoring of the main functions implemented in each of the modules that make up the system. Each module has a set of functional requirements briefly described in the Table 1, presenting the functions performed by the applications of the NutriOdonto environment.

### 2.3. NutriOdonto Data Structure

**Figure 5:**
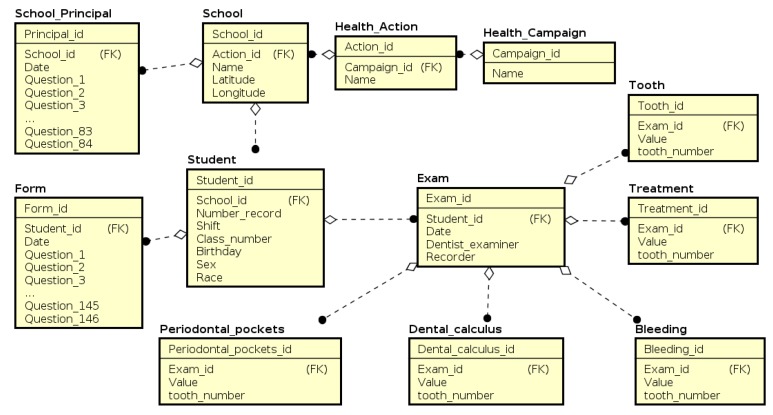
Data structure for storing information collected via the NutriOdonto environment mobile application. Source: Author’s own creation.

The entity and relationship model that represents the data structures of the NutriOdonto environment is described in Figure 5. These are all attributes fulfilled during the data collection process for the formation of the database for the epidemiological survey. The processing of exploratory analysis, inferences and other studies were conducted according to the data presented in this diagram. The starting point of the model is the health campaign data structure. It represents a broader set of activities in which health actions are performed. A campaign can be carried out over a longer period of time, for example annually. Health actions are small activities that together are incorporated into a given campaign. In this context, a campaign can be, for example, an epidemiological survey in the state of Tocantins, while the actions are occasional surveys conducted in the municipality.

For each action, it is necessary to register the schools where the survey will be performed. As previously mentioned, the research actions were carried out from the “Health at School” Program, characterizing the school units as the environment for the research data collection. An important element is the registration of the school’s geographical location as a geographical point that helps in analyzing the region’s information once it is understood that the school unit serves students from the region. For each school, a unit characterization survey is conducted through a questionnaire applied to the principal. The questionnaire is applied on a certain date and is accompanied by several questions of characterization of the school unit. After registering the school and the principal, the student’s registration data is recorded, such as date of birth, gender, race, among others. Importantly, student identification information is not stored in the database in order to ensure the anonymity.

The student data structure is the starting point for epidemiological survey data. From that, it is possible to complete the student questionnaire by means of the form structure, and associate it with the dental exams, represented by the exam data structure. A dental exam consists of two complementary structures, crown and treatment, responsible for storing the corresponding value of each type of answer that can be noted on the form. They constitute the odontogram. It should be noted that it is not possible to record null values in the attributes of an exam. Standard fill values have been set by entering the value ’0’ for healthy (wholesome) teeth. Table 2 presents the codes used to fill in the odontogram.

Completing the oral examination data from the table guidelines, allows the registration of the characterization of decayed, extracted and filled teeth of each student. This process allows the counting of codes for stipulation of the DMF index, which numerically describes the average prevalence of caries in the permanent teeth of a population group. Such information is of paramount importance to public health managers since it provides a general context of the treatment needed to serve that population. This set of information allows an exploratory analysis to be performed in order to quantify and qualify the stored data using visualization mechanisms. The data management mechanism of epidemiological research makes the process of data analysis more efficient as it utilizes computational means of storing, retrieving and processing digital information effectively since computational processing minimizes human error problems by increasing quality of stored data, and effective because it makes the data and analysis information accessible for the long term and still reproducible.

(1) $$CPO_{i}=x+y+z+w,i=1,\cdots ,N$$

(2) $$CPO_{avg}=\frac{\sum_{i=1}^{N}CPO_{i}}{N},i=1,\cdots ,N$$

(3) $$CPO_{\%}=\frac{CPO_{i}*100}{N},i=1,\cdots ,N$$

(4) $$ceo_{i}=x+y+w,i=1,\cdots ,N$$

(5) $$ceo_{avg}=\frac{\sum_{i=1}^{N}ceo_{i}}{N},i=1,\cdots ,N$$

(6) $$ceo_{\%}=\frac{ceo_{i}*100}{N},i=1,\cdots ,N$$

The calculation of the ceo and the CPO indicated in Equations (1) and (4) is performed for each of the *N* students who underwent the epidemiological exams, in which the *X* value determines the number of decayed teeth or surfaces per person, the *Y* value shows the number of crowned teeth or surfaces per person, value *Z* refers to the number of teeth or surfaces with extractions per person, and value *W* indicating the the number of teeth or surfaces with extractions indicated per person. This set of individual information allows a broader epidemiological analysis, for example, by obtaining the average index of a school unit or municipality, for instance, Equation (2) presents the average DMF index. Epidemiological indexes may be associated with other attributes such as gender, age, and so on.

### 2.4. Research Execution

**Figure 6:**
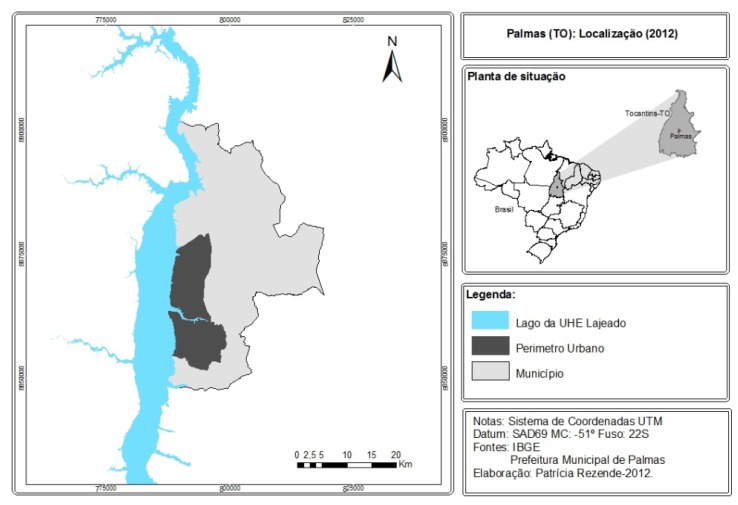
Map with the location of Palmas/TO. Source: Professor João Aparecido Bazolli [21].

This research was conducted in the form of a cross-sectional, analytical study and quantitative approach. The study was conducted in the city of Palmas, capital of the state of Tocantins, in municipal schools that have the 9th grade of elementary school. Shown in the Figure 6, the city of Palmas-TO has a Human Development Index (HDI) of 0,788 and is located in the northern region of Brazil. It has an area of 2219 km^2^, an estimated population of 299,127 people and a demographic density of 102.9 inhab/km^2^ [18].

In 2013, the city of Palmas/TO had twenty-four schools participating in the School Health Program (PSE). Among these, six belonged to the state network and eighteen to the municipal school system. In 2017, the municipality jumped to 104 schools with adherence to the PSE, of which 32 schools belonged to the state network and 72 to the municipal school system. Of all 72 municipal schools, 56 had elementary school education, and 26 of those schools had students enrolled in 9th grade [16]. In August 2017, the total number of students enrolled in these 26 schools was 16,321, of which 2014 were in 9th grade of the traditional elementary school, as it is shown in Table 3. The population cut in 9th grade students was adopted by the following facts:

- The last year of elementary school was considered a coherent parameter for evaluation of the reflexes of exposure to public policies focused on adolescents, and the Brazilian educational system predicts a grade-school adjustment in which ideally, students between the ages of 13 and 15 should attend the 9th grade of elementary school [17];
- The study of dental caries in adolescents is not very common, both in Brazil and in other countries (including developed ones), leaving a gap in the oral health knowledge of this population segment [19];
- Although the age of 12 is recommended by the WHO and its CPO-D is used as an international standard for the evaluation of dental caries, our study privileged the age of 15, as it is also important since its supposed increase in relation to the age of 12 indicates the degree of severity of the disease [20];
- The 9th graders have a level of education that allows them better reading and comprehension for use of self-administered questionnaire [17].

This study included all the municipal schools of Palmas-TO with students enrolled in the 9th grade of elementary school and their respective students, of both genders, enrolled in this grade. The schools were represented by their respective principals, who participated in the survey by answering the questionnaire with the profile of the respective schools. The following were excluded from this study: (a) students who for some reason (medical certificate, maternity leave, etc.) were not in school on the day of the questionnaire application and/or for the oral examination; (b) students who refused to sign the Consent Form (TA) and their parents or legal guardians refused to sign the Free and Informed Consent Form (TCLE); (c) students enrolled in the Youth and Adult Education teaching modality (EJA), excluded for being out of the school leaving age.

Data collection was performed through an application run by electronic devices (smartphones/Tablets/Computers). For the development of the application, an Epidemiological Survey Form, it was adopted as instruments; a self-administered structured questionnaire for the students’ socioeconomic characterization; and a Self-administered Structured Questionnaire for sociodemographic characterization of Palmas municipal elementary schools. The questionnaire applied to the students was elaborated with 146 questions, subdivided into 12 classes of questions about characteristics, behaviors and habits that result in a good quality of oral and nutritional health. Table 4 presents the questionnaire’s classes of questions, the details of the information obtained from the questionnaire classes applied in the school units, and the questions associated with each classes.

The data collection environment was developed to insert the data obtained in the oral exams and in the two questionnaires applied, respectively, to the students and the principals of the schools involved in the study, according to Table 5. The oral examination was performed on adolescents with an average age of 15, enrolled in the 9th grade of elementary school, from Palmas municipal schools, with the objective of knowing the prevalence of dental caries in the researched group. For the assessment of the dental condition of these students, ceo-d (deciduous dentition) and CPO (permanent dentition) indexes were adopted as proposed by the WHO, which express the sum of decayed, lost and filled teeth divided by the number of individuals surveyed [4]. The epidemiological inquiry was conducted by applying a questionnaire and performing oral examinations, both with the filling completed in applications installed on mobile devices. Before completing the questionnaires and taking the exams, students were contacted and informed about the benefits, adverse effects of the oral examination, the need to sign the free Informed Consent terms (TCLE) and the Terms of Consent (TA). After clarifying all the ethical implications of the research, students were invited to participate in the epidemiological inquiry.

Each oral examination team consisted of two dental surgeons: an examiner and an annotator, both wearing gloves, masks, aprons, and caps—All disposable. In the exams number 5 mouth mirrors and wooden spatulas were used. Performed under natural light, on student shifts and in the school yard or in adapted rooms,—All examinations were preceded by the distribution of brushes and toothpaste donated by the researcher. Notes were posted directly to the applications.

The Primary Care Oral Health Teams (ESB) of the Palmas municipal health network were trained for the epidemiological survey, between April and September of 2018, in workshops and conversation circles, whose purpose was to detail the operationalization of work stages, to clarify the attributions of each participant and discuss theoretical and practical aspects of the indexes that should be used. It is noteworthy that the WHO establishes standards and diagnostic criteria for oral health epidemiological studies and recommends that all examiners involved in these studies should previously participate in a calibration, ie, training that involves repetition of examinations in the same people by the same examiners or by the same examiner at different times, so as to select professionals who are able to reproduce in a statistically reliable manner, the criteria adopted in the study, in order to reduce interpretation discrepancies in diagnoses [4,5]. In addition to the researcher, 16 other dental surgeons linked to seven Palmas Community Health Centers (CSC) were trained. The calibration technique adopted was the consensus [22,23], calculating the coefficients of agreement between each examiner and the results obtained by the team’s consensus. The model proposed by the WHO [5] was taken as a reference and, for this purpose, the weighted Kappa coefficient was calculated for each examiner, age group and grievance studied, with a value of 0.65 as the minimum acceptable limit [15].

Data from the epidemiological survey were collected using mobile devices and analyzed using computational techniques based on statistical analyses. After completion of the field teams’ work, the files were transferred to servers for general analyses processing, through estimates of averages, prevalence and the respective standard errors, calculated using the “Complex Samples” module from the Statistical Package for Social Science (SPSS) program, which considers the planning variables and inclusion of the basic weights resulting from the sampling process [24].

## 3. Results

The results are expressed as mobile and web applications for the collection and exploratory analysis of epidemiological data. Both applications adopt the Creative Commons Non-Commercial and Attribution License (BY-NC), allowing licensees to copy, distribute, display and run the software, as well, to develop works derived from it, provided that licensees provide credit to authors and use applications for non-commercial purposes. The software is the result of a research project that received logistical support from Palmas City Hall, Palmas School of Public Health Foundation (FESP), Federal University of Viçosa and Federal University of Tocantins. The public repository with NutriOdonto source code can be found at https://github.com/bcunhasa/nutriodonto. The following subsections present some functions and interfaces of mobile and web applications.

### 3.1. NutriOdonto Mobile App

**Figure 7:**
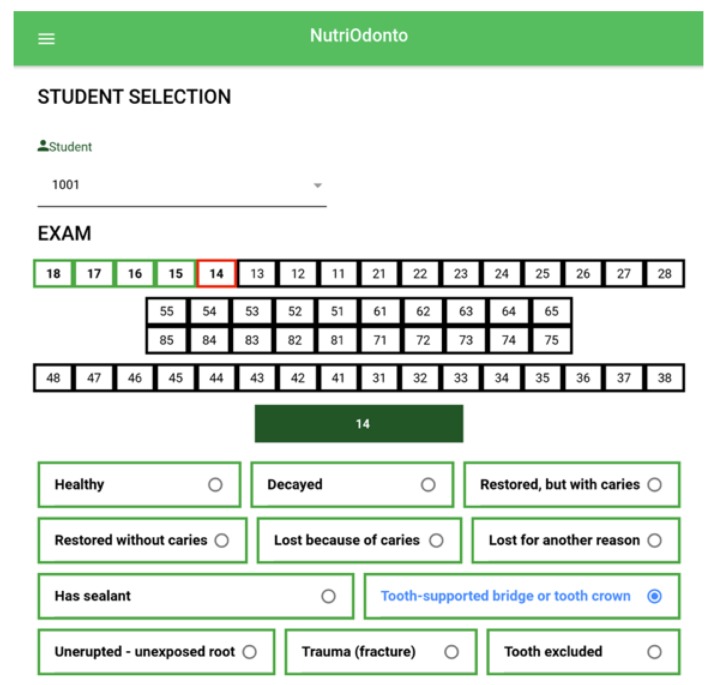
Odontogram of the mobile application. Source: Figure elaborated by the own author.

Figure 7 presents the interface of the odontogram data collection mechanism in the NutriOdonto application. Initially, the note taker selects the student to take the exam by means of the secret identification key. Then, as the dentist performs the exam, the note taker marks tooth by tooth what the situation evaluated is. It can be noted that there are 11 states, as defined in the odontogram reference table. Following the example in the figure below, it is possible to verify that the code 14 tooth was selected and the bridge or crown support status is assigned. The dental chart can be completed through the mobile app and also through the NutriOdonto portal, being thus the first solution to make collection more efficient. The advantage of the filling in in the portal is due to the registration directly into the NutriOdonto environment database, as all information registered in the portal is stored directly in the database. In the case of the application, information is recorded on the mobile device for a later transfer to the central database.

Filling by means of the application makes the activity more efficient, as in order to define the state of a tooth, it only takes two taps on the screen, one to indicate which tooth is analyzed and the other to indicate the state the tooth is in. tooth. The registration process becomes more effective as the recorded information will be properly stored in an electronic means, in several information stores, in the local and the central database and in backup systems, making it possible to efficiently retrieve information for performing diverse processing. In addition to that, the information collected and recorded in databases allows greater efficiency in the processing of analyses, which are important for the execution of statistics and decision-making.

### 3.2. Data Collection Application

**Figure 8:**
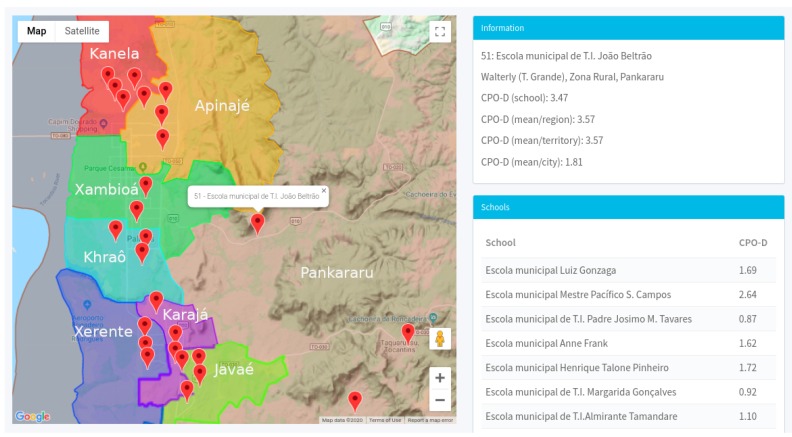
Geographic map of the location of schools in health territories with the respective calculated CPO-d. Source: Figure elaborated by the own author.

A computational environment was developed for the automated realization of the process of collection and formation of the database from the questionnaires applied to the students and principals of the municipal schools. The environment consists of a mobile application, developed for remote data collection in schools, and also an internet information system for synchronization and management of all data obtained in the field collection. The management system can be accessed through a web browser (http://nutriodonto.iacuft.org.br/). The system presents the geographical location of all schools that have data collection actions that are previously planned and the ones that have been visited, and consequently, had questionnaires applied as well as data collected. Figure 8 presents a system-generated map of school locations with planned visits for questionnaire application and data collection.

The environment relates the school units with the health territories of the city, named Apinajé (yellow), Javaé (light green), Kanela (red), Karajá (purple), Khraô (light blue), Xambioá (green), Xerente (blue) and Pankararu (brown), as shown in Figure 8 [25]. The intersection of locations highlights the health area in which the schools are included and consequently identifies which health units serves the students of the schools inserted in that health territory. This visualization allows relating the information obtained in the data collection for the execution of data crossing, helping thus, in the analysis of the actions’ impact in that community. The municipality of Palmas currently has 85 Family Health Strategy teams, of which 71 with Dental Teams, distributed in 33 existing Health Centers. It is estimated that currently, more than 80% of the population, the equivalent of approximately 234,000 people, is covered by Oral Health actions [26].

**Figure 9:**
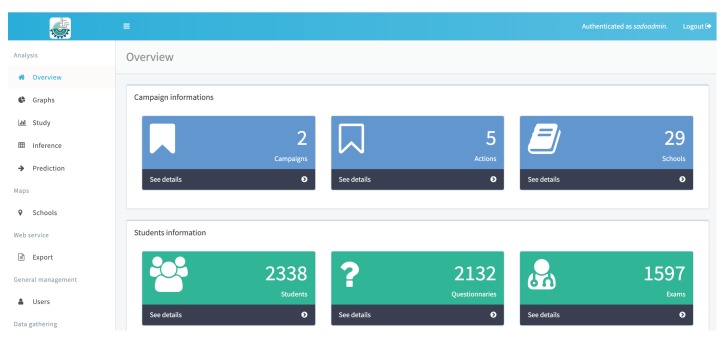
Desktop with overview of the web application. Source: Figure elaborated by the own author.

The Figure 8 presents the results of the oral exams processing, collected through the odontogram presented in the Figure 7, which performs the analysis of the epidemiological survey showing the CPO-D value identified in the school as the average of the CPO-D in the health region where the school is located and also the overall average CPO-D identified for the municipality of Palmas. The computing environment owns restricted access, therefore, in order to view the collected data, it is necessary that a registered researcher enter the username and password granted to him/her to access the desktop and the functionality of the system. When accessing the environment, a work area is presented, as shown in Figure 9, containing the information about the research conducted (campaigns), the number of schools visited, the number of students who answered the survey, with the number of questionnaires answered and exams performed.

**Figure 10:**
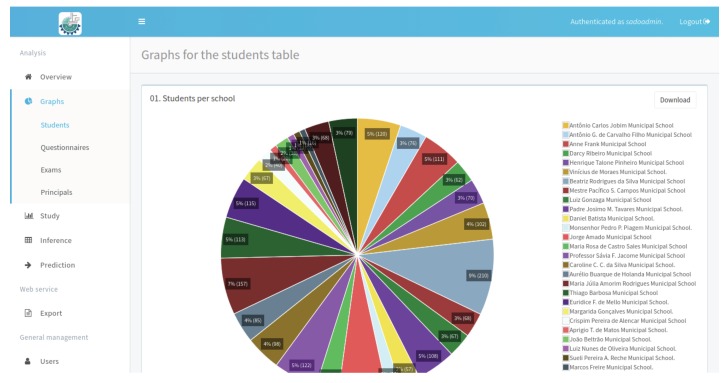
Graphs with the quantity of students by schools. Source: Figure elaborated by the own author.

**Figure 11:**
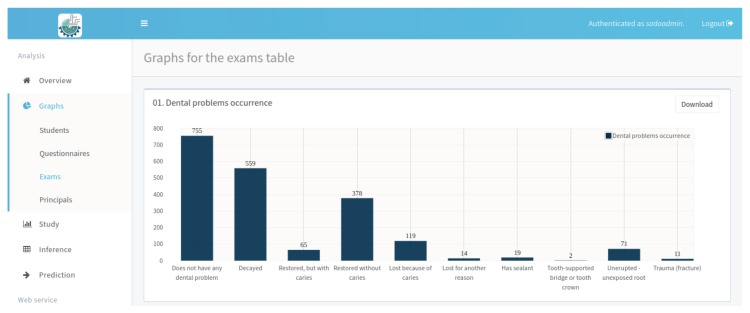
CPO and ceo-d indexes results. Source: Figure elaborated by the own author.

Figure 10 presents the report with the percentage of student participation in the survey by school. Note that the day of the oral examination may differ from the day of the completion of the questionnaire, which is why the number of questionnaires and the number of exams is different, ie the number of students enrolled in the system does not correspond to the number of exams or questionnaires answered. The report presented in the Figure 11 generated by NutriOdonto shows the exploratory analysis result obtained through the processing of the students questionnaires.

**Figure 12:**
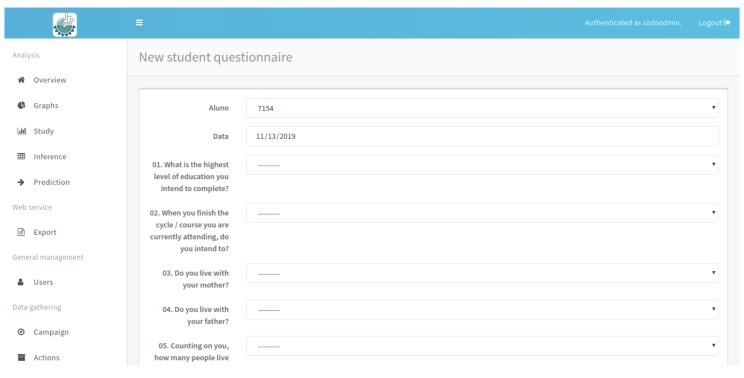
Questionnaire fragment applied to student’s. Source: Figure elaborated by the own author.

The computational environment was developed in way to allow the research to be extended to other school units in several municipalities. For this purpose, the system allows the registration of campaigns, which identifies the macro action search to be performed in a certain period of time, then defines the actions scheduled within a campaign, and in the end, determines the locations that will be incorporated to a particular action in a campaign. The ”Campaign Info” section, shown in Figure 9, presents the sets of information related to search planning. The student questionnaire, proposed in the methodology and implemented in the mobile application has a fragment of the questions presented in Figure 12. The student receives a randomly generated code that enables him to fill in the data, eliminating the possibility of identification in order to preserve anonymity.

## 4. Discussion

Currently, new Information and Communication Technologies (ICT) are being widely used in healthcare to assist in decision-making [27], in the management of health problems and in the expansion of the perspective of service provision in this sector [28].

This study has developed a software (NutriOdonto) for the collection, registration and data storage from oral health epidemiological research. In addition to replacing the use of CPO Index paper sheets proposed by Klein and Palmer with electronic forms, the system presented, among other features, the exemption of the data transfer step recorded for a computer [29], thus contributing to the reduction of information distortion, generating more reliable and faster data [30]. In addition to the data collection obtained by performing the CPO Index, NutriOdonto enabled the application of two structured electronic questionnaires, whose purposes were to know the individual, behavioral and socioeconomic characteristics of the students surveyed, as well as the sociodemographic characteristics of the schools involved. The spread of mobile application use in health care was studied by Da Silva Bonome [31]. These researchers found that in 2011, Brazil broke record sales of smartphones, reaching the mark of 9 million devices sold and it is estimated that in 2012 this number would reach the mark of more than 15 million smartphones sold. Indeed, several Institutional Design methods/procedures have been developed in the last decades aiming at the construction of technological tools that has been improving the performance of its users in the most diverse sectors of social life [11].

In the health domains, the development of these new technologies has revolutionized the clinic and its routines, as well as the form of relationship between users of health systems and professionals of the area [8], including with enormous potential to impact the cost for health services [32]. According to Pereira [33], publications in the health applied mobile technology research line is a new and growing field due to the popularization of smartphones and tablets. However, in a literature review of the electronic databases Pubmed, Scielo, BVS and Google Scholar, using the descriptors: software, epidemiological inquiry, CPO Index and oral health in English, Spanish and Portuguese, we found an insignificant number of studies published addressing the development of mobile technologies for oral health epidemiological surveys.

Similarly, research analyzing the content and usability of mobile oral health applications found that dental application studies are comparatively small and that only a limited number of screening and diagnostic applications have been developed for research, practice management, dental education and scheduling [32]. In Brazil, research similar to the one that developed NutriOdonto was conducted by Sundefeld and Gotlieb [34]. These researchers presented a computer system, called ICADPLUS, developed for database elaboration, data tabulation, CPO index calculation and statistical analysis for confidence interval estimation and comparison of results of two populations. At that time the main feature offered by the system was the dismissal of specialized professionals in the field of dentistry and computing in order to transfer data, requiring the operators the minimum typing knowledge.

Another automated system for collecting data for oral health epidemiological survey called the Health Data Collection Program (PCDS) was developed by De Barros Lima [30]. In addition to the proven effectiveness in data collection, the ease of handling, the increase in the validity of the study, as well as the agility of database construction were also found. The study, however, evidenced the need to develop computer systems that overcome the limitations of PCDS, such as: limitation of data storage capacity, need for different programs for the final availability of data in a format that is compatible with the statistical program, among others.

In Thailand, researchers from the Community Dentistry Department at Bangkok’s Chulalongkorn University School of Dentistry, have developed another Oral Health Research Mobile App similar to PCDS and NutriOdonto. Called OHSMA. The App is designed specifically for dentists and oral care professionals, allowing its users to skip the data transfer step recorded on paper forms, to reduce the time and cost of the data entry step as well as the possible errors that may occur during this step, especially when using large amounts of information sets [29].

Although the internet already makes free software available that performs several functions similar to those of NutriOdonto, one of its main advantages is that it works as a tool that uses standard forms developed and tested by Brazilian public health institutions, for which NutriOdonto will be freely available. In addition, it is an application developed for data collection in the field without the need for it to be necessarily connected to the world wide web (internet).

Thus, when impossible to connect to the internet at the time of the epidemiological investigation, the application temporarily stores the information collected in a database suitable for smartphones or tablets, to later send them to a central base, enabling the collection of information in remote environments, a fundamental requirement for conducting epidemiological surveys digitally, in regions of difficult access and economically poor, such as the Brazilian Legal Amazon, where the municipality of Palmas, capital of the State of Tocantins, is located. Another advantage is the possibility of reproducibility of the studies, since the data are stored in digital means, in a structured database, it is possible to apply the data analysis methodology under the data set, using the tools developed for the purpose of the epidemiological study, as well as third-party methods and tools under the data.

The security of data collected and stored by NutriOdonto received special attention from researchers. Applying encryption that all information and data collected are encrypted before being inserted into the databases, and only being retrieved in their entirety through the holder of the private key for decrypting the information. This care directly benefits system users by preventing them from serious repercussions in case the security and privacy of the information obtained is violated [35]. In addition, the NutriOdonto environment works with geographic information tools obtained from the collection of geographic coordinates at the time of completing the epidemiological questionnaire in school units. These tools enabled the analysis of the information correlating it with the location where the epidemiological research was performed.

The geographic information complementing the information from the epidemiological studies allowed the identification of the characterization of each region, in terms of oral health, population profile, eating habits, among others. Thus, it is possible to compare, for example, socioeconomic data between different regions, drawing thematic maps on different aspects of studies, comparing eating behaviors, using health services in terms of eating habits, how the profile of a school unit is, from one region compared to the school unit in another region totally opposite the first. These data can be compared with the ones obtained from other sources of information, such as data from the Brazilian Institute of Geography and Statistics (IBGE), Basic Education Development Index (IDEB), Human Development Index (HDI), as well as data from National or Regional Oral Health Surveys conducted by the Brazilian government and other federative entities.

The environment has the potential to expand the questionnaires used in the epidemiological inquiry, allowing the insertion, update or removal of new questions for this one or others. Data are organized by campaigns to make data collection actions flexible across different regions, dates, participants, as well as incorporating a set of actions into a given campaign, creating small actions that are key components of a broader action, resulting in a grouping of small actions. The environment also allowed the data to be organized by year, by region, by action, and the researcher had to make a previous planning and proceed with the release of the data in the central application, according to his execution plan of actions.

NutriOdonto can receive new structures of epidemiological survey forms allowing the expansion of the modalities of epidemiological surveys, which currently includes survey on dental caries and periodontal examinations, as it also has tools for conducting exploratory studies on the data, being thus possible the construction of mechanisms of analysis and inference, as well as the generation of graphs and tables, simultaneously conducting the surveys themselves, that is, generating and storing data and information in real time, if connected to the internet. In Brazil, one of the main objectives of the inclusion of the Oral Health Team in the Family Health Strategy (FHS) was to improve oral health indicators and increase the population’s access to oral health actions [36,37]. For this reason, oral health epidemiological surveys are fundamental and indispensable.

However, in our country, the unsatisfactory financing of health in general and oral health in particular is notorious. High expenditures on input/equipment costs and people management are considered barriers that hinder the management of health services [37]. NutriOdonto therefore presents itself as a mobile device with the potential to alleviate health burdens [35]. Thus, in the specific case of a municipality like Palmas, a tool such as NutriOdonto, designed to conduct epidemiological surveys with the range of functionalities presented, can make a difference and contribute to the reorganization of dental practice, with the evaluation of programs and impact of oral health policies, as well as the incorporation of health surveillance proposals that contemplate the implementation of the doctrinal principle of the Unified Health System (SUS), the integrality of care, guaranteed by the Brazilian Federal Constitution of 1988.

Finally, it should be emphasized that, despite the reported difficulties with the financing of oral health, epidemiological surveys cannot and should not be understood as sporadic or optional actions by dental managers and professionals, since epidemiology is paramount for planning, monitoring. 483 and evaluation of public health services [38].

## 5. Conclusions

The development of NutriOdonto contributed decisively to the promptness in collection, registration, storage and especially in the construction of the database and respective analyses the remote databases synchronized with the centralized databases, information was updated and results of the analyses were processed instantly, yielding results, including graphs, figures and tables in real time. On the other hand, the replacement of paper forms with electronic forms minimized possible typos, reduced the use of cellulose paper and financial costs, streamlined the work process, in addition to contributing to several aspects related to the development of the study.

Despite the fact that the internet already makes a free software available, with many functions similar to the ones performed by NutriOdonto, most of the results obtained by the developed system, were very encouraging, distinguishing it including, from other pre-existing ones, such as: (i) collecting digital data, in the field and in regions of difficult access, without necessarily being connected to the internet; (ii) having been developed in partnership with Brazilian public institutions for which the software will be made available, free of charge, -with significant potential to impact the evaluation of oral health programs and policies in different government levels; (iii) to enable the correlation of the information obtained with the location where the epidemiological research was carried out and the identification and characterization of each researched region, a functionality that is very important for studies in the Amazon region.

New research need to be carried out to perfect and/or improve the performance of the developed system. We believe that our study contributes as an incentive to conduct oral health epidemiological surveys in the most diverse communities.

## Figures and Tables

**Table 1:** Modules functional requirements.

**Module: Portal**
RF0001: Record Registration Information: Registration, update and exclusion of news, photos and movies record of epidemiological survey of actions undertaken during the campaigns to document the field activities. It is a set of mechanisms that help to share information about the actions taken by the epidemiological survey team.
**Module: Administration**
RF0010: Record Users: Registration, update and exclusion of system users. Includes queries by name present a list of users registered.
RF0011: Manage Users and Roles: Registration, update and exclusion of system users roles defining user access rules to the environment and data stored in the system database Includes queries by role name present a list of roles registered in the system.
**Module: Integration**
RF0012: Export Data Records: This module is responsible for exporting database data to external systems through web services. The export is done through XML or JSON files allowing the manipulation of the data by other external tools.
**Module: Data Gather**
RF0002: Manage campaign (Record epidemiological research action): Registration, update and exclusion of campaign data in the system. Includes queries by name present a list of registered campaign. A campaign is a way of classifying major events for epidemiological survey, for example, held annually.
RF0003: Manage actions (Record epidemiological research action): Registration, update and exclusion of actions data in the system. Includes queries by name present a list of registered actions. It is a way of classifying small events within an epidemiological survey campaign
RF0004: Record schools: Registration, update and exclusion of school data in the system through the management of school name and geolocation information. The schools are included in a previously registered action and are plotted on a thematic map of the locations served in an epidemiological survey campaign. Includes queries by school name present a list of registered schools.
RF0005: Record principal school: Registration, update and exclusion of school data characterization in the system by completing the director’s questionnaire. Includes queries by school name present a list of registered actions. In this case the principal should answer the questionnaire based on the characteristics of the school. The principal questionnaire has 84 questions.
RF0006: Record student: Registration, update and exclusion of student registration data in the system through information on gender, date of birth and race. The student reports secret registration code, school and class. Includes queries by secret registration code present a list of students associated with their respective school.
RF0007: Answer student registration questionnaire: Registration, update and exclusion of student questionnaire from secret registration code, school and class. The student reports their code in the mobile or web environment and completes the questionnaire. The environment preserves student anonymity through an encrypted key (secret code).
RF0008: Answer student epidemiological questionnaire: Registration, update and exclusion of student epidemiological data present in the questionnaire. The student enters the secret registration code and initiates the completion of the questionnaire by selecting the options presented that are most appropriate to his or her behavioral profile. The epidemiological questionnaire has 145 questions. Includes queries by secret registration code present a list of students associated with their respective school.
RF0009: Perform oral exam: Registration, update and exclusion of student epidemiological exam. The filling is performed by a note taker from the dentist information. In this case, the note taker requests the student’s secret code to be filled in and begins to fill in the forms from the dentist’s instructions. Two exams are performed: dental caries and need for treatment and periodontal condition.
**Module: Analysis**
RF0013: Generate Graphs: Function responsible for presenting a set of statistical analyzes from the data collected by the campaigns and epidemiological survey actions. It is a visualization mechanism that performs exploratory analyzes to characterize and summarize the data collected by the teams. The informations are classified based on campaigns, actions and time parameters.
**Module: Geographical**
RF0014: Manage Geographic Information: Function related to the management of information about geolocation of school units, campaigns and actions. It treats information under location parameters to assist visualization of statistical data on the spatial data layers relating health and education information. This module enhances the visualization of analyzes under a geographic component allowing to evaluate the data under the region in which the actions were performed.

**Table 2:** Odontogram with two complementary structures – crown and treatment. Source: Author’s own creation.

Deciduous Dentition (Caries)	Permanent Dentition (Caries)	Treatment
A: Healthy	0: Healthy	0: None
B: Has Caries	1: Has Caries	1: Restoration of 1 surface
C: Restored, but with caries	2: Restored, but with caries	2: Restoration of 2 or more surfaces
D: Restored without caries	3: Restored without caries	3: Crown for any reason
E: Lost because of caries	4: Lost because of caries	4: Aesthetic facet
F: Lost for another reason	5: Lost for another reason	5: Pulp treatment and restoration
G: Has dental sealant	6: Has dental sealant	6: Extraction
H: Tooth-supported bridge	7: Tooth-supported bridge	7: White spot remineralisation
K: Unerupted/unexposed root	8: Unerupted/unexposed root	8: Sealant
T: Trauma (fracture)	T: Trauma (fracture)	9: No information
L: Not considered	9: Not considered	-

**Table 3:** Palmas-TO Municipal Schools that have the 9th grade of Elementary School. Total number of students enrolled, number of students enrolled in 9th grade of Elementary School (Traditional) in 2017. Source: Table created by the author himself from data obtained from the Municipal Secretary of Education of Palmas/TO [16].

Healthy Territory	Schools	Total of Students	Total of Students in 9th Grade
Krahô	Escola Municipal Antonio Carlos Jobim	599	99
Xambioá	Escola Municipal AntonioG. De Carvalho Filho	618	70
Apinajé	Escola Municipal Anne Frank	838	80
Xambioá	Escola Municipal Darcy Ribeiro	476	117
Apinajé	Escola Municipal Henrique Talone Pinheiro	794	80
Xambioá	Escola Municipal de Tempo Integral Vinícius de Moraes	483	81
Kanela	Escola Municipal Beatriz Rodrigues da Silva	897	157
Kanela	Escola Municipal Mestre Pacifico S. Campos	431	74
Kanela	Escola Municipal Luiz Gonzaga	264	65
Kanela	Escola Municipal Tempo Integral Padre Josimo M. Tavares	1087	116
Apinajé	Escola Municipal Tempo Integral Daniel Batista	386	29
Apinajé	Escola Municipal Tempo Integral Monsenhor Pedro P. Piagem	466	35
Javaé	Escola Municipal Jorge Amado	568	159
Javaé	Escola Municipal Maria Rosa De Castro Sales	703	79
Krahô	Escola Municipal Professora Savia F. Jacome	558	71
Javaé	Escola Municipal de Tempo Integral Caroline C. C. Da Silva	1187	72
Karajá	Escola Municipal Aurelio Buarque De Holanda	776	115
Xerente	Escola Municipal Maria Julia Amorim Rodrigues	694	176
Karajá	Escola Municipal Thiago Barbosa	896	104
Xerente	Escola Municipal de Tempo Integral Euridice F. De Mello	1088	68
Xerente	Escola Municipal de Tempo Integral Margarida Gonçalves	1028	62
Pankararu	Escola Municipal Crispim Pereira Alencar	565	33
Apinajé	Escola Municipal de Tempo Integral Aprigio T. De Matos	235	15
Pankararu	Escola Municipal de Tempo Integral Joao Beltrao	217	21
Pankararu	Escola Municipal de Tempo Integral Luiz Nunes De Oliveira	249	22
Pankararu	Escola Municipal de Tempo Integral Sueli Pereira A. Reche	218	14
		**16.321**	**2.014**

**Table 4:** Classification of the questions from the questionnaire applied with the students. Source: Table created by the author himself from the National School Health Survey conducted in Brazil in 2015 [17].

Class	Question Description	Questions
Personal characteristics	General information about academics (preserving anonymity)	1–18
Nutritional data	Information on student behavior, frequency and eating habits.	19–62
Physical activities	Information about the physical activities performed, characteristics and frequency.	63–72
Smoking	Information on smoking by the student or a close person	73–81
Alcohol consumption	Information Alcohol consumption by the student or or a close person	82–89
Drug use	Information about illicit drug use by the student or close people	90–95
Personal relationships	Information on relationship with parents or guardians and with schoolmates.	96–107
Hygiene and oral health	Information on personal hygiene and oral health.	108–113
Security	Information on security that impedes attendance in the school unit and diverse assaults	114–130
Access to health services	Information on access, frequency, care, quality of use and health services.	131–138
Body image	Information about body image, overweight, medication or dietary supplements.	139–145
Your opinion	Students are asked to express their opinion by evaluating the questionnaire.	146

**Table 5:** Instruments used in the study development. Source: Chart adapted by the author himself from the National School Health Survey conducted in Brazil in 2015 [17].

Group	Instrument	Objective	Author
**Oral exam**	CPO-D index	To evaluate the prevalence of dental caries in permanent dentition.	Klein and Palmer (1937)
	ceo-d index	To evaluate the prevalence of dental caries in primary dentition.	Gruebbel (1944)
**Socioeconomic and Behavioral Characteristics of Students**	Questionnaire of the Students	Describe the socioeconomic and behavioral characteristics of the students.	Adapted from IBGE (2016)
**Structural and Demographic Characteristics Of Schools**	Questionnaire of the school Principal	Describe the structural and sociodemographic characteristics of the schools surveyed.	Adapted from IBGE (2016)
